# Predicting the drop out from the maternal, newborn and child healthcare continuum in three East African Community countries: application of machine learning models

**DOI:** 10.1186/s12911-023-02305-1

**Published:** 2023-09-25

**Authors:** Chenai Mlandu, Zvifadzo Matsena-Zingoni, Eustasius Musenge

**Affiliations:** School of Public Health, University of Witwatersrand, Johannesburg, South Africa

**Keywords:** Continuum of Care, Maternal Newborn and Child Healthcare, Machine learning

## Abstract

**Background:**

For optimal health, the maternal, newborn, and child healthcare (MNCH) continuum necessitates that the mother/child receive the full package of antenatal, intrapartum, and postnatal care. In sub-Saharan Africa, dropping out from the MNCH continuum remains a challenge. Using machine learning, the study sought to forecast the MNCH continuum drop out and determine important predictors in three East African Community (EAC) countries.

**Methods:**

The study utilised Demographic Health Surveys data from the Democratic Republic of Congo (DRC) (2013/14), Kenya (2014) and Tanzania (2015/16). STATA 17 was used to perform the multivariate logistic regression. Python 3.0 was used to build five machine learning classification models namely the Logistic Regression, Random Forest, Decision Tree, Support Vector Machine and Artificial Neural Network. Performance of the models was assessed using Accuracy, Precision, Recall, Specificity, F1 score and area under the Receiver Operating Characteristics (AUROC).

**Results:**

The prevalence of the drop out from the MNCH continuum was 91.0% in the DRC, 72.4% in Kenya and 93.6% in Tanzania. Living in the rural areas significantly increased the odds of dropping out from the MNCH continuum in the DRC (AOR:1.76;95%CI:1.30–2.38), Kenya (AOR:1.23;95%CI:1.03–1.47) and Tanzania (AOR:1.41;95%CI:1.01–1.97). Lower maternal education also conferred a significant increase in the DRC (AOR:2.16;95%CI:1.67–2.79), Kenya (AOR:1.56;95%CI:1.30–1.84) and Tanzania (AOR:1.70;95%CI:1.24–2.34). Non exposure to mass media also conferred a significant positive influence in the DRC (AOR:1.49;95%CI:1.15–1.95), Kenya (AOR:1.46;95%CI:1.19–1.80) and Tanzania (AOR:1.65;95%CI:1.13–2.40). The Random Forest exhibited superior predictive accuracy (Accuracy = 75.7%, Precision = 79.1%, Recall = 92.1%, Specificity = 51.6%, F1 score = 85.1%, AUROC = 70%). The top four predictors with the greatest influence were household wealth, place of residence, maternal education and exposure to mass media.

**Conclusions:**

The MNCH continuum dropout rate is very high in the EAC countries. Maternal education, place of residence, and mass media exposure were common contributing factors to the drop out from MNCH continuum. The Random Forest had the highest predictive accuracy. Household wealth, place of residence, maternal education and exposure to mass media were ranked among the top four features with significant influence. The findings of this study can be used to support evidence-based decisions in MNCH interventions and to develop web-based services to improve continuity of care retention.

## Introduction

In the era of the Sustainable Development Goals (SDGs), reducing the global burden of preventable maternal, newborn, and child mortality and morbidity is a top priority [1, 2]. Despite gains in maternal and child health during the Millennium Development Goals (MDGs) era, over 2.7 million mothers and newborn babies died in 2017 [3, 4]. Sub-Saharan Africa (SSA) alone accounted for 66% (196 000) of maternal deaths, 39% of neonatal deaths (999 000) and infant deaths in 2017 [3, 4]. Most maternal and neonatal deaths occur due to avoidable complications and illnesses during pregnancy and childbirth [5, 6].

The concept of the Continuum of Care (CoC) has been brought to light to enhance Maternal, newborn and child healthcare (MNCH) through integrated service delivery (1, 7). An effective CoC connects critical MNCH packages throughout the pregnancy, delivery, and postpartum stages. Completing the MNCH CoC helps achieve the SDG 3 goals by reducing severe maternal and neonatal morbidity rates, mortality rates, and long-term physical and psychological complications (8). For instance, antenatal care (ANC) visits can identify and treat problems during pregnancy and increase the mother’s chances of receiving appropriate care at birth [9–11]. Skilled care during labour and delivery ensures safe and healthy delivery and reduces the risk of death for both the mother and baby [12]. Postnatal care (PNC) is also recommended at birth and extends up to six weeks to avoid postpartum haemorrhage and other causes of maternal and neonatal mortality [13]. A lack of appropriate care at any stage of the CoC will lead to poor MNCH outcomes.

Studies conducted in East Africa including Kenya and Tanzania showed that only 10–34% of the women received complete packages of maternal health care services [14, 15]. Factors such as place of residence, maternal education, maternal age, parity, household wealth, media exposure, travel distance and mode of transport have been factors found to be associated with the drop out from the CoC [8, 14–19]. Knowledge from these studies can be applied to develop machine learning (ML) models that can predict the likelihood of a mother/child not completing the continuum of MNCH and identify predictors with significant influence on discontinuity of care. This information will aid in the development of targeted interventions to improve MNCH retention.

Previously conventional analytical approaches such as logistic regression models have commonly been used to analyse maternal healthcare utilisation data [20]. However, ML methods such as the Decision Tree and Random Forest have the potential to outperform conventional statistical methods because of various attributes including the ability to handle large and non-linear complex data, non-reliance on prior assumptions and multiple interactions between predictors [20, 21]. Thus, ML methods have been reported to produce better fitting models than conventional logistic regression models [20–22]. Although ML methods have better prediction performance than conventional statistical methods when applied to large datasets, studies on the application of ML methods in SSA countries remain uncommon [20, 21]. Thus, this study aimed to predict the likelihood of a mother/child dropping out from the MNCH continuum by applying reliable ML predictive models and determining the most influential predictors in three EAC countries including the DRC, Kenya and Tanzania.

## Methodology

### Study design and settings

This study utilised secondary data from the last rounds of Demographic Health Survey (DHS) surveys in the DRC (2013/14), Kenya (2014) and 2015/16 Tanzania (2015/16) DHS surveys. The DRC, Kenya and Tanzania are neighbouring countries in the EAC [23, 24].

### Study population

The study comprised reproductive women aged 15–49 years who delivered their children in the past 5 years preceding DHS surveys in the DRC, Kenya, and Tanzania. Only women who attended at least one ANC visit and gave responses on skilled birth attendance (SBA) and PNC were included in the study, and only information concerning the last birth in the last 5 years was used.

### Data source and sampling

The DHS is a nationally representative survey of household samples that provides comprehensive information on the population and health including MNCH. The DHS utilises multistage sampling, where the first stage involves the selection of enumeration areas (EAs) or clusters drawn from census files. The second stage involves the random selection of individual households within each selected EA or cluster and the probability of selection of each household differs from cluster to cluster. The analysis used total sample sizes of 8,545, 6,432 and 6,664 reproductive-age women and children born in the past five years preceding each country’s DHS survey in the DRC, Kenya and Tanzania respectively. The combined total sample for the three countries was 21,641.

### Measurement of variables

The dependent variable in this study was drop out from the MNCH continuum. Antenatal care drop out was considered if a woman had less than four ANC visits during her most recent pregnancy. Skilled birth attendance drop out was considered if a woman had four or more ANC visits but did not receive SBA (delivery was not assisted by healthcare professionals, i.e., midwives, nurses, doctors, and/or health officers). Postnatal care drop out was considered if a woman received SBA but did not attend PNC with the child within the first 6 weeks of delivery. The drop out from the MNCH continuum was coded as 1 if a woman/child drops out of ANC, SBA, and PNC and 0 if otherwise [8].

The independent variables considered for analysis included demographic and socioeconomic variables. The demographic and socioeconomic variables included the place of residence (rural/urban), mother’s current age group (15–24 years/40–44 years/25–49 years), mother’s level of education (no education/primary, secondary and tertiary), birth order (1,2,3 or more), relationship status (no current partner/ has a current partner), exposure to mass media (no, yes), access to money for medication ( no big problem, big problem ), travel distance for medication (no big problem, big problem), household size (< 4/ ≥5), household head (male/ female) and household wealth (poor/middle/rich), and household wealth status, which was grouped into tertiles (poor, middle and rich) in this study, using the household wealth index variable (poorest, poor, middle, richer and richest) in the DHS surveys data [25].

### Statistical analysis

Using the STATA package, data cleaning was performed to prepare the dataset for analysis. The analysis was adjusted for sampling survey weights. The univariate analysis was conducted to describe women’s characteristics. Bivariate analysis was conducted to assess the women’s characteristics by dependent and independent variables and the chi-square was used to test the differences. The multivariate logistic regression models were fitted to identify the factors associated with the drop out from the MNCH continuum. In the multivariable analysis, adjusted odds ratios (AOR) with 95% confidence intervals (CI) were used to assess the significance of the relationship between the dependent variables and the independent variables.

ML predictive models were built and trained in Python 3.0 using combined DHS surveys data for the three countries. Predictors that were found significant in the multivariable logistic regression analysis were used in the ML analysis. The ML analysis utilised the classification method. The datasets were randomly assigned to the training and testing datasets using an 80/20% split. The training data consisted of the data used to develop the models and the test data or validation sets were used for evaluating the performance of the models [22].

Five classification algorithms namely the Logistic Regression, Random Forest, Decision Tree, Support Vector Machine (SVM) and Artificial Neural Network (ANN) were employed. In this study, the dependent variable (drop out from the MNCH continuum) classes were disproportionate. Most machine learning algorithms work best when the number of samples in each class is about equal because most algorithms are designed to maximize accuracy and reduce errors. Thus, random oversampling was conducted to balance the distribution of classes of the drop out from the MNCH continuum. Random oversampling involves supplementing the training data with multiple copies of some of the minority classes. In this study, K-means Synthetic Minority Oversampling Technique (SMOTE) was employed to correct the class imbalance [26].

The performance of the ML predictive models was assessed using predictive accuracies including Accuracy, Precision, Recall, Specificity, F1 score, as well as the AUROC. The ranking of features was conducted on the ML model with better performance using the feature importance permutations technique. This technique breaks the relationship between the feature and the target. The drop in the model score shows how much the model depends on the feature [27].

## Results

Table 1 shows the characteristics of women in the three countries under study. Most women in the DRC (64.2%), Kenya (60.2%) and Tanzania (69.9%) lived in rural areas. Over two-thirds of the women in the DRC (69.8%), Kenya (70.8%) and Tanzania (67.4%) were aged between 25 and 49 years. Most of the women in the DRC (85.0%), Kenya (81.7%) and Tanzania (80.7%) had a current partner. A greater proportion of the women had primary or no education in the DRC (56.5%), Kenya (63.1%) and Tanzania (83.6%). Women with birth order of three or more were about 11% in the DRC (10.6%), 4% in Kenya (4.2%) and 5% in Tanzania (4.7%). A greater proportion of the women in Kenya (79.6%) and Tanzania (67.0%) were exposed to mass media, whilst a greater proportion of the women in the DRC (87.1%) were not exposed to mass media.

A greater proportion of women in the DRC (70.2%) and Tanzania (52.4%) experienced big problems with access to money for medication, on the other hand a greater proportion of women in Kenya (60.9%) did not experience problems with access to money for medication. The majority of the women in the DRC (60.7%), Kenya (75.2%) and Tanzania (54.9%) experienced big problems with travel distance for medication. Most households in the DRC (80.0%), Kenya (68.9%) and Tanzania (81.4%) were led by males. The majority of the women in the DRC (76.6%), Kenya (58.8%) and Tanzania (73.0%%) had households with 5 or more members. Over one-third of the women in the DRC (39.3%), Kenya (37.5%) and Tanzania (41.6%) were from poor-income households.

**Table 1:** Characteristics of women in countries under study

Variables	DRC N(8,545) * n(%)	Kenya N(6,432) * n(%)	Tanzania N(6,664) *n(%)
**Place of residence**			
Urban	3,083(35.8)	2,524(39.8)	2,004(30.1)
Rural	5,539(64.2)	3,820(60.2)	4,663(69.9)
**Maternal age-group**			
Young women (15–24 years)	2,607(30.2)	1,849(29.2)	2,175(32.6)
Older women (25–49 years)	6,015(69.8)	4,495(70.8)	4,492(67.4)
**Relationship status**			
Has no current partner	1,295(15.0)	1,161(18.3)	1,287(19.3)
Has current partner	7,327(85.0)	5,183(81.7)	5,380(80.7)
**Maternal education**			
Primary and below	4,870(56.5)	4,005(63.1)	5,573(83.6)
Secondary and above	3,752(43.5)	2,339(36.9)	1,093(16.4)
**Birth order**			
One	3,998(46.4)	4,392(69.2)	4,232(63.5)
Two	3,711(43.0)	1,690(26.6)	2,120(31.8)
Three or more	913(10.6)	262(4.2)	315(4.7)
**Exposure to mass media**			
No	7,497(87.1)	1,296(20.4)	2,201(33.0)
Yes	1,115(12.9)	5,048(79.6)	4,465(67.0)
**Access to money for medication**			
No big problem	2,568(29.8)	3,867(60.9)	3,176(47.6)
Big problem	6,052(70.2)	2,474(39.1)	3,491(52.4)
**Travel distance for medication**			
No big problem	5,228(60.7)	4,769(75.2)	3,658(54.9)
Big problem	3,391(39.3)	1,573(24.8)	3,009(45.1)
**Household head**			
Male	6,895(80.0)	4,373(68.9)	5,429(81.4)
Female	1,727(20.0)	1,971(31.1)	1,238(18.6)
**Household size**			
4 or less	2,021(23.4)	2,617(41.3)	1,803(27.0)
5 or more	6,601(76.6)	3,727(58.8)	4.864(73.0)
**Household wealth**			
Poor	3,391(39.3)	2,379(37.5)	2,772(41.6)
Middle	1,724(20.0)	1,180(18.6)	1,258(18.9)
Rich	3,507(40.7)	2,784(43.4)	2,636(39.5)

* n(%) weighted counts and proportions of the variable

### Patterns of the drop out from the MNCH continuum

**Fig. 1:**
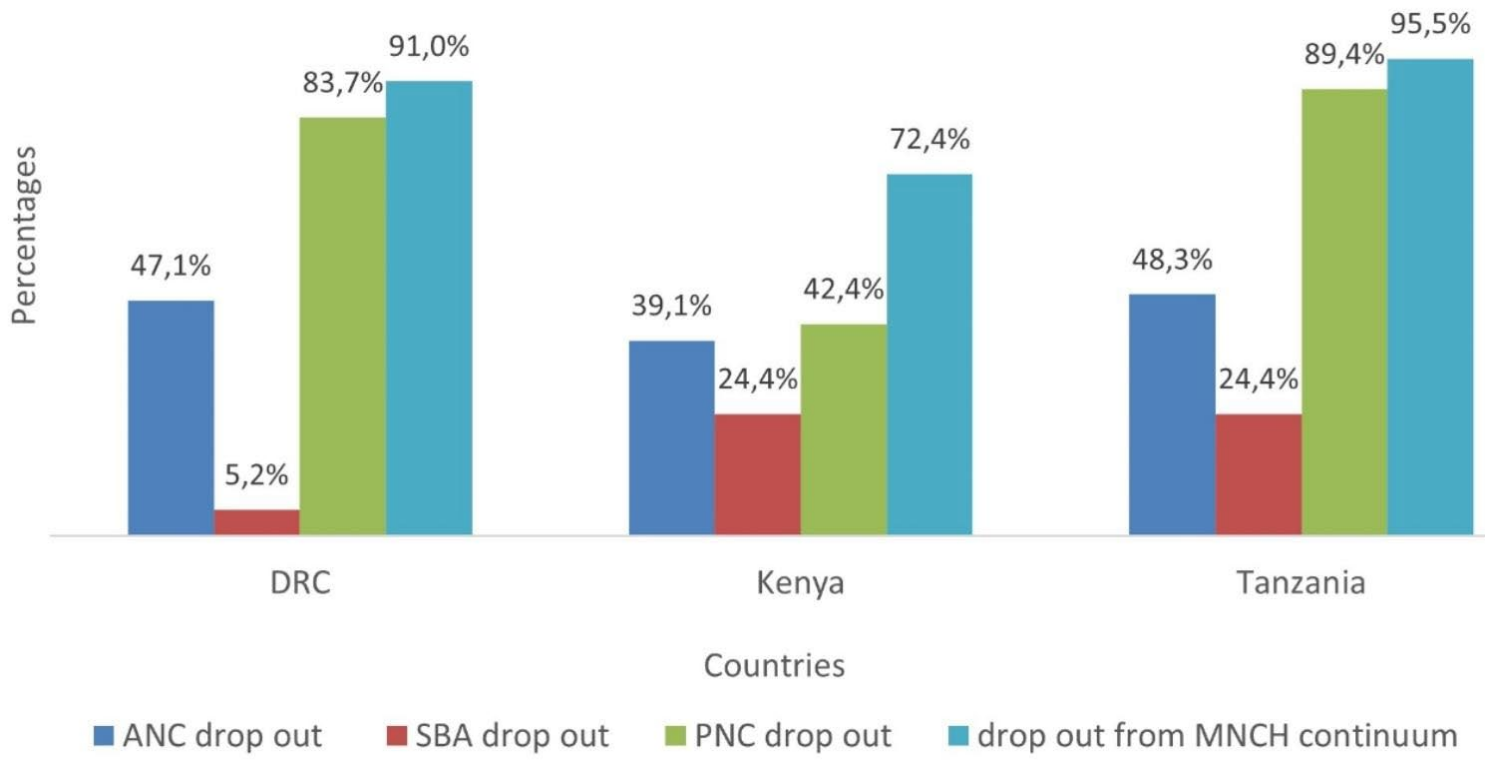
Patterns of the drop out from the MNCH continuum among reproductive-age women in the last 5 years preceding each country’s DHS survey

Figure 1 compares the drop out from the MNCH continuum across the three countries under study. The largest gap and contributor to the drop out from the CoC occurred during the postpartum period in the DRC (83.7%), Kenya (42.4%) and Tanzania (89.4%). The overall drop out from the MNCH continuum was very high across the three countries, with proportions of 91.0% in the DRC, 72.4% in Kenya and 95.5% in Tanzania.

Table 2 shows results of the factors associated with the drop out from the MNCH continuum in the DRC, Kenya and Tanzania. The place of residence, mother’s education and exposure to mass media were common factors significantly associated with the drop out from the MNCH continuum across the three countries. The study showed that living in rural areas, having a lower education, and having no exposure to mass media was positively associated with the drop out from the MNCH continuum.

Further, the influence of factors such as maternal age group, birth order, access to money for medication, travel distance for medication and household size varied by country. The findings showed that the odds of dropping out from the MNCH continuum was significantly lower among older women aged 25–49 years in the DRC (AOR:0.74;95%CI:0.57–0.95) and Kenya (AOR:0.81;95%CI:0.66–0.98). An increase in birth order was also significantly associated with dropping out from the MNCH continuum in the DRC and Kenya.

The study also found that women who experienced big problems with access to money for medication (AOR:1.23;95%CI:1.03–1.46) and travel distance for medication (AOR:1.25;95%CI:1.02–1.52) had significantly increased odds of dropping out from the MNCH continuum in Kenya. Women belonging to large households also had significantly higher odds of dropping out from the MNCH continuum in Kenya (AOR:1.45;95%CI:1.22–1.72). It was also observed that women from poor-income and middle-income households had significantly increased odds of dropping out from the MNCH continuum in Kenya and Tanzania.

**Table 2:** Factors associated with the drop out from the MNCH continuum in the DRC, Kenya and Tanzania

	DRC			Kenya			Tanzania		
	*n(%)	Univariate OR(95%CI)	Adjusted OR(95%CI)	*n(%)	Univariate OR(95%CI)	Adjusted OR(95%CI)	*n(%)	Univariate OR(95%CI)	Adjusted OR(95%CI)
**Place of residence**									
Urban	2,633(85.4))	Reference	Reference	1,599(61.8)	Reference	Reference	1,836(91.6)	Reference	Reference
Rural	5,213(94.1)	2.73(2.20–3.39)**	1.76(1.30–2.38)**	3,033(79.4)	2.39(2.04–2.79)**	1.23(1.03–1.47)**	4,532(97.2)	3.17(2.39–4.19)**	1.41(1.01–1.97)**
**Maternal age-group**									
Young women (15–24 years)	2,396(91.9)	Reference	Reference	1,378(74.6)	Reference	Reference	2,084(95.8)	Reference	Reference
Older women (25–49 years)	5,450(90.6)	0.85(0.67–1.07)	0.74(0.57–0.95)**	3,212(71.5)	0.86(0.721–1.01)	0.81(0.66–0.98)**	4,282(95.4)	0.90(0.67–1.22)	0.89(0.66–1.20)
**Relationship status**									
Has current partner	6,672(91.2)	Reference	Reference	3,761(72.6)	Reference	Reference	5,218(95.3)	Reference	Reference
No current partner	1,172(90.5)	0.94(0.70–1.25)	1.05(0.76–1.45)	831(71.6)	0.95(0.78–1.16)	0.98(0.78–1.23)	1,240(96.4)	1.30(0.89–1.90)	1.50(0.97–2.32)
**Maternal education**									
Secondary and above	4,620(86.0)	Reference	Reference	1,400(79.7)	Reference	Reference	987(90.3)	Reference	Reference
Primary and below	3,225(95.0)	3.03(2.44–3.75)**	2.16(1.67–2.79)**	3,192(59.9)	2.63(2.25–3.09)**	1.56(1.30–1.84)**	5,381(96.6)	3.02(2.24–4.10)**	1.70(1.24–2.34)**
**Birth order**									
One	3,550(88.0)	Reference	Reference	3,002(68.3)	Reference	Reference	4,006(94.7)	Reference	Reference
Two	3,446(92.9)	1.64(1.31–2.05)**	1.51(1.19–1.90)**	1,365(80.7)	1.94(1.62–2.33)**	1.38(1.14–1.68)**	2,056(97.0)	1.81(1.30–2.53)**	1.34(0.94–1.91)
Three or more	850(93.1)	1.66(1.14–2.52)**	1.59(1.06–2.37)**	226(86.2)	2.90(1.82–4.62)**	1.72(1.06–2.79)**	307(97.5)	2.21(0.88–5.57)	1.37(0.55–3.40)
**Exposure to** **mass media**									
Yes	926(83.1)	Reference	Reference	3,491(69.2)	Reference	Reference	4,215(94.4)	Reference	Reference
No	6,908(92.2)	2.39(1.85–3.08)**	1.49(1.15–1.95)**	1,101(85.0)	2.52(2.08–3.06)**	1.46(1.19–1.80)**	2,153(97.8)	2.64(1.85–3.79)**	1.65(1.13–2.40)**
**Access to money** **for medication**									
No big problem	2,271(88.4)	Reference	Reference	3,299(69.2)	Reference	Reference	2,992(94.2)	Reference	Reference
Big problem	5,573(92.1)	1.53(1.23–1.89)**	1.18(0.94–1.46)	1,291(82.1)	1.92(1.64–2.24)**	1.23(1.03–1.46)**	3,376(96.7)	1.79(1.35–2.39)**	1.24(0.90–1.71)
**Travel distance** **for medication**									
No big problem	4,722(90.3)	Reference	Reference	2,612(67.5)	Reference	Reference	3,467(94.8)	Reference	Reference
Big problem	3,121(92.0)	1.24(0.99–1.55)	0.92(0.73–1.15)	1,978(79.9)	2.04(1.71–2.44)**	1.25(1.02–1.52)**	2,902(96.4)	1.49(1.10–2.01)**	0.99(0.70–1.41)
**Household head**									
Male	6,268(90.9)	Reference	Reference	3,153(72.1)	Reference	Reference	5,180(95.4)	Reference	Reference
Female	1,578(91.4)	1.06(0.82–1.37)	1.09(0.82–1.45)	1,439(73.0)	1.05(0.89–1.23)	0.96(0.80–1.15)	1,189(96.0)	1.16(0.81–1.67)	1.06(0.0.72–1.56)
**Household size**									
4 or less	1,839(91.0)	Reference	Reference	1,673(64.0)	Reference	Reference	1,693(93.9)	Reference	Reference
5 or more	6,007(91.0)	1.00(0.78–1.29)	1.05(0.79–1.40)	2,919(78.3)	2.03(1.74–2.37)**	1.45(1.22–1.72)**	4,675(96.1)	1.60(1.20–2.15)**	1.18(0.86–1.61)
**Household wealth**									
Rich	3,198(94.3)	Reference	Reference	1,663(59.7)	Reference	Reference	2,422(91.9)	Reference	Reference
Middle	1,597(92.6)	1.88(1.37–1.59)**	1.05(0.76–1.44)	910(77.1)	2.27(1.85–2.78)**	1.49(1.20–1.86)**	1,219(96.9)	2.74(1.86–4.03)**	1.67(1.07–2.60)**
Poor	3,051(87.0)	2.48(1.94–3.18)**	0.94(0.66–1.35)	2,019(84.9)	3.78(3.18–4.48)**	1.89(1.53–2.33)**	2,728(98.4)	5.44(3.78–7.83)**	2.89(1.84–4.54)**

*n% is weighted counts and proportions** is a significant p-value < 0.05

### Predictive modelling

**Fig. 2:**
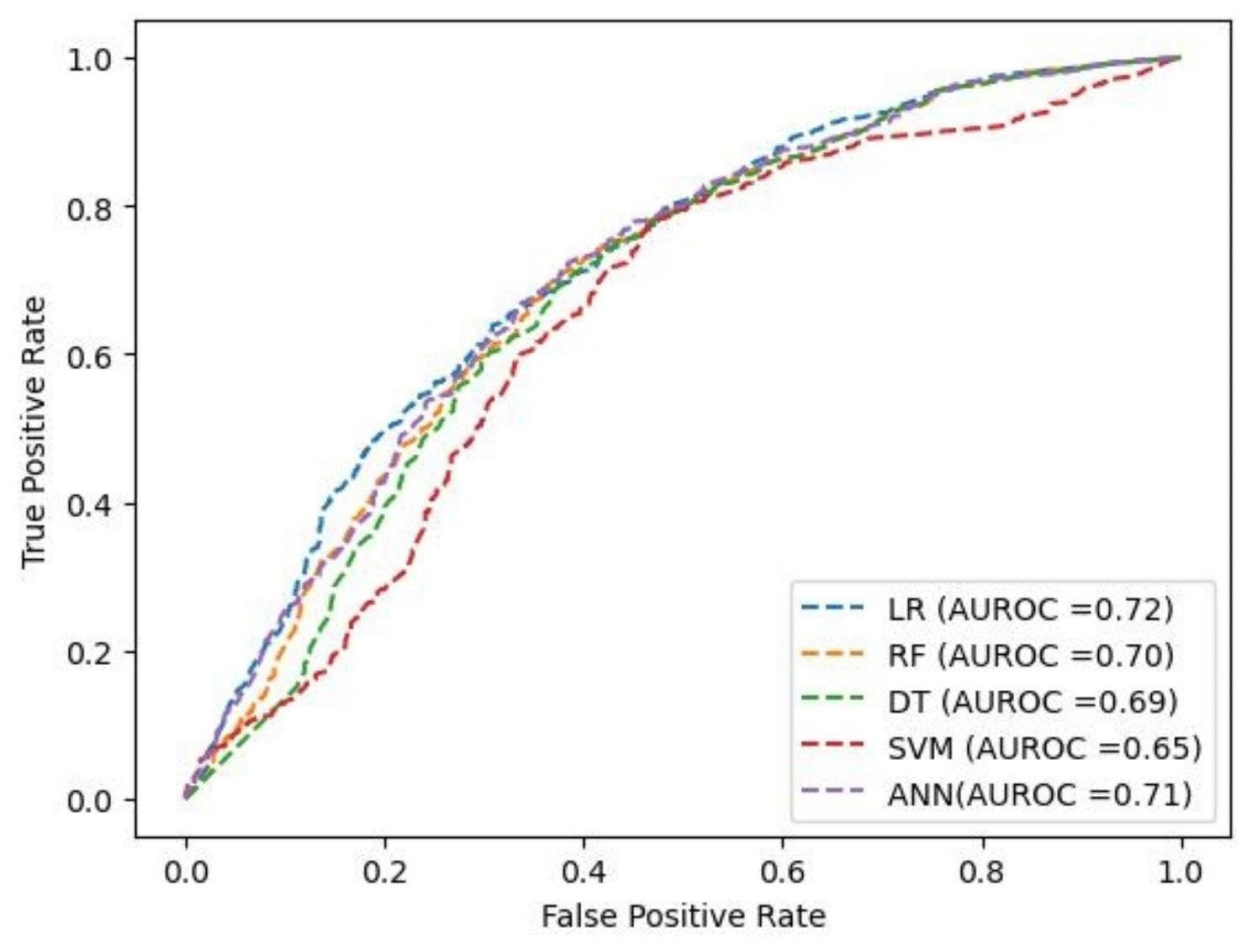
AUROC parameters for the five ML classification models

**Fig. 3:**
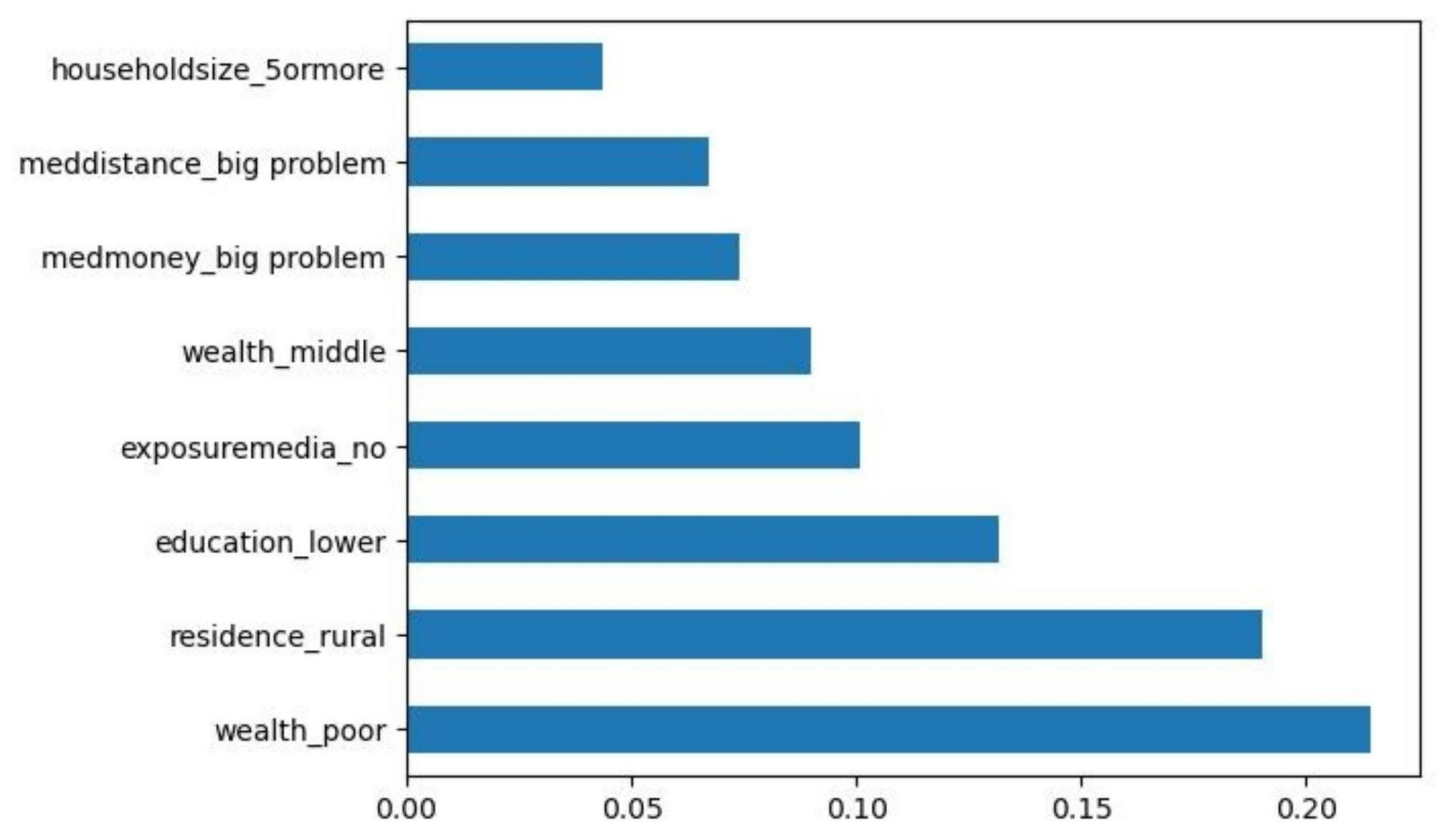
Feature (variable) ranking using the Random Forest

Among all the ML prediction models, the Random Forest had better prediction performance based on the model prediction accuracies. The model performed at an accuracy of 75.7%, implying that among the 30,404 instances (after using K-means SMOTE), the model correctly classified 23,016 instances. Of the total 23,016 instances, the model had Precision of 79.1%, Recall of 92.1%, Specificity of 51.6%, and an F1 score of 85.1% (Table 3). The AUROC was 70% (Fig. 2). Household wealth, place of residence, maternal education and exposure to mass media were the top four most influential predictors of the drop out from the MNCH continuum (Fig. 3).

**Table 3:** Prediction analysis of the drop out from the MNCH continuum using combined DHS surveys data for the DRC, Kenya and Tanzania (2013–2016)

Algorithm	Accuracy	Precision	Recall	Specificity	F score
Logistic regression	74.3	77.2	92.2	53.5	84.0
Random Forest	75.7	79.1	92.1	51.6	85.1
Decision tree	75.2	78.6	92.0	51.6	84.8
SVM	75.3	78.8	91.9	51.0	84.8
ANN	75.4	78.5	92.3	53.3	84.8

## Discussion

The present study illustrated the determinants of the drop out from the MNCH continuum and developed ML models to forecast the drop out from the MNCH continuum using nationally representative DHS survey data from three EAC countries including the DRC, Kenya, and Tanzania. The study findings showed that most women dropped out from the continuum of MNCH across the three EAC countries. The prevalence of the drop out from the MNCH continuum was 91.0% in the DRC, 72.4% in Kenya and 93.6% in Tanzania. The largest gap and contributor to the high drop out from CoC was observed at PNC. The prevalence of the drop out from the MNCH continuum in the present study was consistent with other studies [7, 28]. The high drop out in the MNCH continuum poses a higher risk of maternal and neonatal morbidity and mortality to many women and children due to missed opportunities for interventions in the CoC [14].

The study found that rural dwellers were significantly more likely to drop out from the MNCH continuum in the DRC, Kenya and Tanzania. These findings have been reported in other studies, where rural women were less likely to complete the CoC (7, 29, 30). These results are consistent with the view that rural women may encounter higher obstacles to obtaining maternal healthcare than urban women due to constrictive cultural norms, long travel distances, unaffordability of medicine, and the burden of caring for larger families (7, 14). Additionally, our analysis indicated that dropping out of the MNCH continuum, particularly in Kenya was linked with unaffordable medicine, long travel distances, and larger households. These factors may represent potential obstacles for rural Kenyan women’s retention in the CoC [31, 32]. Scaling up community education programs and developing policies that improve access to health facilities and supply and affordability of drugs could improve the retention of rural women in the continuum of MNCH [33].

The study revealed that low maternal education attainment was a positive predictor of dropping out from the MNCH continuum in the DRC, Kenya and Tanzania. Previous studies support our findings, the studies found that less educated women were more likely to be retained in maternity care [7, 18]. Better education is believed to be an essential factor in creating better awareness and increasing knowledge of the importance of MNCH. Thus, government policies that promote female education and community interventions such as media campaigns would increase the awareness of women on the importance of the continuum of MNCH [34]. The role of mass media was also evident in determining retention in MNCH as cited in previous research [35]. Women who were not exposed to mass media were more likely to drop out of the MNCH continuum in the three countries. These results demonstrate the crucial role of the media in promoting and raising awareness about the continuation of MNCH. Thus, governments and other non-governmental organisations should invest continuously in the design and implementation of MNCH services utilisation educational programs through mass media channels to increase the use of these services [35].

The study also found that older women in the DRC and Kenya were less likely to drop out from the continuum of MNCH. However, there is a lack of consensus on the influence of the mother’s age on maternal health utilisation [29, 36, 37]. Our findings are similar to a previous study in Ghana which found that older women were less likely to drop out from maternal healthcare [36]. This could be the reason that older women aged 25–49 years have gathered immense knowledge on the need to utilise maternal healthcare, which may positively influence their use of these services.

Higher birth order was positively associated with drop out from the MNCH in the DRC and Kenya. These findings corroborate results from other studies [7, 29]. Possibly high parity women place high value on lower pregnancies because of experience in pregnancy and childbirth [38, 39]. The retention of multigravida women in the continuum of MNCH could be enhanced by expanding community education programs through mass media campaigns.

Women in poorer households were more likely to drop out from the continuum of MNCH in Kenya and Tanzania. These findings were consistent with other studies in Kenya and elsewhere [29, 40, 41]. Although MNCH services are being provided for free in Kenya and Tanzania, other factors, such as inadequate healthcare provision and transportation costs, may act as impediments to the complete utilisation of the MNCH continuum among the poor [41–44]. The indirect costs for transportation, medication, and healthcare-related services might have contributed to the differences observed in the drop out from the CoC among the poor and rich women [41–44]. A multi-pronged approach to addressing barriers to accessing care among the poor is required, taking into account other potential barriers such as travel costs to health facilities and a lack of staff or medication [43].

Regarding the ML predictive analysis, this study showed that ML methods predict the drop out from the MNCH continuum better than the conventional logistic regression method. The ML model performance results showed that the logistic regression model had the lowest prediction accuracy compared to other ML classification models. This result is not surprising, since ML methods are documented to outperform conventional logistic methods in several fields of medicine [20, 21]. Our results also showed the Random Forest had the highest prediction accuracy compared to the rest of the models. These results showed that the Random Forest is the most suitable algorithm in this study to accurately predict the drop out from the MNCH continuum. The Random Forest is a commonly used ML model which combines the output of multiple decision trees to reach a single result. It is easily interpretable and flexible as compared to other ML algorithms such as ANN and SVM [45].

Using the Random Forest, the study further ranked the most important predictors associated with the drop out from the MNCH continuum. Household wealth, place of residence, exposure to mass media and maternal education were the top four important predictors. The use of ML analysis can be valuable in identifying the most influential predictors for targeted interventions. This information can accelerate the improvement of the utilisation of the MNCH continuum in the SDG era, as it provides public health programmers and policymakers with cost-effective interventions for time and resource management (46). Rapid response mechanisms such as web-based applications can also be developed by applying ML. For instance, web-based applications can be used to assess the probability of a pregnant woman and unborn child dropping out from the CoC based on the mother’s characteristics [20, 47]. This allows for the provision of targeted interventions to pregnant women at high risk of discontinuing care in real-time and improves retention in the MNCH continuum [20, 47].

### Strengths and limitations

The study analysed factors contributing to the drop out from the MNCH continuum among several countries in the EAC. Thus, highlighting the common driving factors which should be considered when designing policies and interventions aimed at improving retention in the MNCH continuum. The study also developed ML models to forecast the drop out from the MNCH continuum, which is computationally strong when handling big data and can be used to classify certain hidden information that could not be detected by conventional statistical methods. However, the study is subject to several limitations. The main components of MNCH rely on the women’s self-report which are subject to recall bias. Another possible limitation is that additional features that could have contributed to the prediction output were not present. This includes information that was not collected in the surveys such as health service provision features (quality of care and availability of drugs and equipment). The ML method is also novel in the SSA, we did not have enough evidence to compare the findings on the prediction of the drop out from MNCH continuum in the EAC countries with other countries in SSA. The ML analysis did not account for survey weights. This is because most ML methods were built for predictions and not ascertaining relationships,and cannot account for survey weights. However, significant factors from the multivariate logistic regression that accounted for survey weights were used in the ML predictions. Finally, it is important to state that both conventional and ML techniques should be embraced and we should take advantage of their strengths depending on the problem to be solved.

## Conclusions

The prevalence of drop out from the MNCH continuum was 91.0% in the DRC, 72.3% in Kenya and 93.6% in Tanzania. The greatest contributor to the drop out from the continuum of MNCH was between delivery and the postpartum period. Place of residence, maternal education and exposure to mass media were common contributing factors associated with drop out from the MNCH continuum in the EAC countries. Among the developed ML prediction models, the Random Forest had better prediction accuracy. The top four predictors with the greatest influence were household wealth, place of residence, maternal education and exposure to mass media.  The results of these findings can help inform evidence-based decisions in MNCH interventions and can also be used to assist in developing web-based applications that help public health practitioners take preventative action to retain more mothers and children in CoC.

## Data Availability

The data used for this study is available on the MEASURE DHS website (https://dhsprogram.com/data/available-datasets.cfm) and can be accessed with the permissible application.

## Abbreviations

ANC: Antenatal care
AUROC: Area under the Receiver Operating Characteristics Receiver
CoC: Continuum of Care
DRC: Democratic Republic of Congo
DHS: Demographic and Health Survey
EAC: East African Community
MNCH: Maternal, newborn and child healthcare
ML: Machine learning
PNC: Postnatal care
SBA: Skilled Birth Attendance
SMOTE: Synthetic Minority Oversampling Technique
SSA: sub-Saharan Africa
